# 1775. Predictor Factors Of Severe Forms Of Rickettsial Infections

**DOI:** 10.1093/ofid/ofad500.1604

**Published:** 2023-11-27

**Authors:** Fatma Hammami, Makram Koubaa, Khaoula Rekik, Amal Chakroun, Fatma Smaoui, Chakib Marrakchi, Mounir Ben Jemaa

**Affiliations:** Infectious Diseases Department, Hedi Chaker University Hospital, University of Sfax, Tunisia, Sfax, Sfax, Tunisia; Infectious Diseases Department, Hedi Chaker University Hospital, University of Sfax, Tunisia, Sfax, Sfax, Tunisia; Infectious Diseases Department, Hedi Chaker University Hospital, University of Sfax, Tunisia, Sfax, Sfax, Tunisia; Infectious Diseases Department, Hedi Chaker University Hospital, University of Sfax, Tunisia, Sfax, Sfax, Tunisia; Infectious Diseases Department, Hedi Chaker University Hospital, University of Sfax, Tunisia, Sfax, Sfax, Tunisia; Infectious Diseases Department, Hedi Chaker University Hospital, University of Sfax, Tunisia, Sfax, Sfax, Tunisia; Infectious Diseases Department, Hedi Chaker University Hospital, University of Sfax, Tunisia, Sfax, Sfax, Tunisia

## Abstract

**Background:**

Rickettsial infections usually mimic benign viral infection due to similarities in clinical symptoms. However, severe forms and complications might occur changing therfore the prognosis of the disease. We aimed to study the predictor factor of severe forms of rickettsial infections.

**Methods:**

We conducted a retrospective study including all patients hospitalized in the infectious diseases department for rickettsiosis. The diagnosis was confirmed by serologies (seroconversion) and/or positive polymerase chain reaction for *Rickettsia* in skin biopsy. Severe forms included cases with acute respiratory distress syndrome, myocarditis, meningitis, meningoencephalitis, renal failure or septic shock.

**Results:**

We encountered 472 cases among which 98 cases were severe (20.8%). Median age was 43[25-60] years among severe forms and 38[24-53] years among non-severe forms (p=0.132). Maculopapular skin rash (56.7% vs 88% ; p< 0.001), arthralgia (56.1% vs 77.3% ; p< 0.001) and eschar (16.3% vs 27% ; p=0.029) were significantly less frequent among severe forms. Meningeal syndrome (34.7% vs 8.3% ; p< 0.001), vomiting (56.1% vs 41.8% ; p=0.011) and cough (28.6% vs 13.9% ; p=0.001) were significantly more frequent among severe forms. Leukocytosis was significantly more frequent among severe forms (35.7% vs 20.6% ; p=0.002). The optimal cut-off value of white blood cell count for predicting severe forms was 7595/mm^3^, with a sensitivity of 61.9% and a specificity of 59.7%. The area under the curve was 0.622 and the 95% conﬁdence interval ranged from 0.558 to 0.686. Elevated C-reactive protein levels (66.3% vs 65.5%; p=0.879) and accelerated erythrocyte sedimentation rate (36.7% vs 40.1%; p=0.543) were noted among severe and non-severe forms of rickettsiosis, with no significant difference.

**Conclusion:**

The revealing symptoms of severe forms were characterized by the presence of vomiting, cough and meningeal syndrome, in the absence of the typical maculopapular skin rash and eschar. Leukocytosis was more frequent among severe forms of rickettsiosis. The diagnosis of severe forms should be kept in mind in order to promptly initiate the adequate treatment.

**Disclosures:**

**All Authors**: No reported disclosures

